# Mortality convergence in the enlarged European Union: a systematic literature review

**DOI:** 10.1093/eurpub/ckaa038

**Published:** 2020-03-24

**Authors:** Rok Hrzic, Tobias Vogt, Fanny Janssen, Helmut Brand

**Affiliations:** c1 Department of International Health, Maastricht University, Care and Public Health Research Institute, CAPHRI, Maastricht, The Netherlands; c2 Population Research Centre, Faculty of Spatial Sciences, University of Groningen, Groningen, The Netherlands; c3 Prasanna School of Public Health, Manipal Academy of Higher Education, Manipal, Karnataka, India; c4 Max Planck Institute for Demographic Research, Rostock, Germany; c5 Netherlands Interdisciplinary Demographic Institute - KNAW / University of Groningen, The Hague, The Netherlands

## Abstract

**Background:**

The high mortality rates in the European Union (EU) Member States that acceded in 2004 sparked political interest in mortality convergence. Whether mortality is converging in the EU remains unclear. We reviewed the literature on mortality convergence in the post-2004 EU territory as a whole. We also explored whether the study designs influenced the results and whether any determinants of mortality convergence had been empirically examined.

**Methods:**

A systematic literature review was performed. Our search included scientific databases and the websites of international governmental institutions and European demographic research institutes.

**Results:**

We uncovered 94 unique records and included seven studies that reported on 36 analyses. There was marked methodological heterogeneity, including in the convergence measures (beta and sigma convergence). All of the beta convergence analyses found narrowing mortality differentials, whereas most of the sigma convergence analyses found widening mortality differentials. The results are robust to the units of analysis and mortality and dispersion measures. Our results also suggest that there is a lack of evidence on the determinants of mortality convergence in the EU.

**Conclusions:**

There is general agreement that the EU regions and the Member States with high initial mortality rates improved the fastest, but this trend did not lead to overall mortality convergence in the EU. The harmonization of mortality convergence measures and research into determinants of mortality convergence are needed to support future EU cohesion policy. Policy-makers should consider supporting areas that have moderate but stagnant mortality rates, in addition to those with high mortality rates.

## Introduction

After the fall of the Iron Curtain, many central and eastern European countries applied for accession, and finally joined the European Union (EU) during its fifth enlargement (2004–07). Most of these new Member States had higher mortality rates than the old Member States (EU-15),^1^ and lie on the eastern side of Europe’s east-west mortality gap.^2^^,^^3^ Health and mortality convergence have been a key political priority in the post-2004 EU, which has committed substantial resources via the Health Programme, Structural Funds and other mechanisms to support efforts to reduce socioeconomic and geographic health disparities.^4^^,^^5^ It is important for the credibility of the EU enlargement process to establish whether existing integration mechanisms perform effectively by closing gaps in development and welfare, including population health and mortality.

Previous empirical and theoretical work on convergence in the EU focuses mainly on regional economic convergence (e.g. Ref.^6^) while convergence in population health and mortality remains less well characterized. Nevertheless, the questions addressed by both are similar enough for the methods and results of EU regional economic convergence studies to be of interest. The classical econometric approach to measuring convergence differentiates between two measures of convergence, beta and sigma convergence, which represent different aspects of the phenomenon.^7^ Beta convergence occurs when poorer economies grow faster than rich ones, and sigma convergence occurs when the inequality (i.e. dispersion) in income decreases over time.^7^ The studies of EU regional economic convergence uncover a multi-speed Europe with multiple convergence clubs without a trend of overall convergence and find that characteristics of regional economies are determinants of long-term development trajectories.^8^

A central theory of mortality convergence is the vanguard–laggard theory of Vallin and Meslé,^9^ which predicts mortality convergence in the post-2004 EU. The authors postulate that mortality convergence or divergence is a consequence of different speeds in the uptake of medical technologies, public health policies and health behaviours. The theory splits countries into the vanguards, which are the first to develop and implement mortality-reducing innovations; and the laggards, which then catch up with varying speeds. Vallin and Meslé argued that the European mortality gap arose primarily because of the slow diffusion across the Iron Curtain of the innovations that enabled the cardiovascular revolution. This impediment to the diffusion of innovation has since been replaced by the Europeanization of health policy^10^ and the formation of a ‘European healthcare union’.^11^

Whether mortality convergence in the EU actually occurred remains unclear due to an inconsistent geographic scope of existing studies. The literature is composed of broadly three groups of studies. In the first group are the studies that examined mortality convergence in the EU using a comparative case study approach that includes only a few countries at a time (e.g. Ref.^12^^–14^) and were unable to assess mortality convergence in the EU as a whole. In the second group are the studies that consider geographic Europe (including non-EU countries) (e.g. Ref.^15^^,^^16^) the results of which may be driven by the unique mortality conditions of countries outside the EU (e.g. Russia). The third group includes the studies with a comprehensive coverage of the enlarged EU, which seems to be rarer and which are the focus of our review.

The results of convergence studies depend on how the respective researchers conceptualized convergence. Meta-analyses of the economic convergence literature found that the results of studies are sensitive to the choice of underlying assumptions and methods.^17^ Researchers have also used different inequality measures when investigating sigma convergence in the context of national mortality convergence.^18^^,^^19^ Different inequality measures lead to different results, as they measure different aspects of inequality.^20^

In addition to understanding the status of mortality convergence in the EU, having knowledge of its facilitators and barriers is necessary to pursue an effective mortality convergence policy. Studies that compared the mortality convergence of a limited number of EU Member States found that health behaviours, healthcare reforms, healthcare quality and accessibility are important factors in mortality convergence.^12–14^ However, as we mentioned above, their findings are difficult to generalize to the EU as a whole. Studies on a wider sample of geographic Europe have shown that mortality levels are associated with a country’s national income,^21^ social welfare spending^22^ and government policy.^23^ Moreover, the factors associated with the overall level of mortality may not be the same as those that influence its distribution.

### Research aims

The aim of this article is to review the literature on mortality convergence in the enlarged EU (after the 2004 enlargement). In addition to examining whether the literature provides evidence of mortality convergence in the EU, we catalogued the definitions and measures of convergence used in the literature and summarized the determinants of convergence that were already empirically evaluated.

## Methods

We performed a systematic literature review in which we operationalized mortality convergence as convergence in standardized all-cause mortality rates and life expectancy at birth. These outcome measures are readily available and have been shown to be robust in space and time.^24^^,^^25^

### Eligibility criteria

We included studies that aimed to investigate mortality convergence in the post-2004 EU territory. This does not mean that the time scope of these studies was limited to the period after 2004; rather, it means that only the geographic scope of the studies had to encompass the territory of the 25, 27 or 28 EU Member States. Thus, our aim was to include mortality convergence studies of all time scopes, as long as they adhered to the geographic scope. The studies had to investigate convergence in terms of standardized all-cause mortality rates or life expectancy at birth, either sex-specific or aggregated. Finally, the studies had to include an explicit quantitative measure of convergence. As we updated our search in May 2019, we were able to include studies that were published up to that point. While we did not explicitly exclude any publication based on language, we used English keywords in our search. See Supplementary table S1 for a detailed description of the inclusion and exclusion criteria.

### Data sources and search strategy

We employed a three-part search strategy. First, we searched titles, abstracts and keywords of publications in electronic databases of research papers, including the Social Sciences Citation Index (1988–present), the Science Citation Index Expanded (1988–present), the Arts and Humanities Index (1988–present), the Emerging Sources Citation Index (2015–present) and MEDLINE (1950–present) using the Web of Science interface. Our keyword strategy is summarized in Supplementary table S2. Second, we searched the websites of WHO Europe (https://apps.who.int/iris/), EU institutions (https://publications.europa.eu), the Organization for Economic Co-operation and Development (https://www.oecd-ilibrary.org) and a selection of demographic research institutes of EU Member States (Max Planck Institute for Demographic Research in Germany, Netherlands Interdisciplinary Demographic Institute, Centre d’Estudis Demografies in Spain, Wittgenstein Centre for Demography and Global Human Capital in Austria, Centre for Population Change in the UK, Hungarian Demographic Research Institute and Institut national d’études démographiques in France) for reports and working papers with relevant titles. Third, we performed several searches in Google Scholar and screened the first 100 hits of each search for publications with relevant titles.

### Selection procedure

The titles and summaries of the records we found were screened for relevance. The full texts of the remaining records were retrieved, and the final inclusion decisions were made after the texts were scrutinized against the inclusion and exclusion criteria. The first author initially performed the selection. The first, second and fourth authors then discussed the records in question and came to a majority decision in case of uncertainty.

### Synthesis

We decided against performing a meta-analysis due to the heterogeneity of the methods used in the included studies. Instead, we provide a structured synthesis.^26^

We examined the main characteristics of the included studies (table 1) and have summarized their findings in relation to their design characteristics in figure 2. The characteristics we focused on include time and geographic coverage, outcome measures, convergence concept, dispersion measures and potential determinants of convergence controlled for in the models. We chose this set of characteristics because meta-analyses of economic convergence literature show these to be the key design aspects underlying heterogeneity of results. The study characteristics were extracted from the methods sections of the included reports. The first author initially performed the extraction. The first, second and fourth authors then discussed the reports in question and came to a majority decision in case of uncertainty.

**Table 1:** Characteristics of included studies

Study	Geographic scope	Temporal scope	Outcome measures	Units of analysis	Convergence concept(s) included	Dispersion measure	Results
Stańczyk,^34^ 2016	260 regions (NUTS 2) in 28 EU Member States (excluding outlier regions)	2002–12	LEB	NUTS 2 regions	Beta convergence	*Not applicable*	Convergence
Maynou and Saez,^31^ 2016	271 regions (NUTS 2) in 27 EU Member States (excluding Croatia)	1995–2011	LEB and all-cause SMR	NUTS 2 regions	Beta and sigma convergence	Coefficient of variation	Both beta convergence analyses: convergence Both sigma convergence analyses: divergence
Maynou et al.,^29^ 2015	271 regions (NUTS 2) in 27 EU Member States (excluding Croatia)	1995–2007	LEB	NUTS 2 regions	Beta and sigma convergence	Coefficient of variation	Convergence
Richardson et al.,^32^ 2014	129 mainland regions (NUTS 2) in 13 EU Member States (Austria, Belgium, Spain, Finland, France, Italy, Portugal, Sweden, Czech Republic, Estonia, Hungary, Lithuania and Poland)	1991–2008	Sex-specific LEB	Deciles based on life expectancy and income	Sigma convergence	Interdecile range	Persistence
Jaworska,^28^ 2014	265 regions (NUTS 2) in 28 EU Member States	2002–12	LEB	NUTS 2 regions	Beta convergence	*Not applicable*	Convergence
Marmot et al.,^30^ 2013	28 EU Member States and 268 EU regions (NUTS 2)	2002–09, 2000–10	Sex-specific LEB	Member States, NUTS 2 regions	Sigma convergence	Range, ratio and Gini coefficient	Member States (2000–10), female LEB, Gini coefficient: convergence Other 13 analyses show persistence
Spinakis et al.,^33^ 2011	27 EU Member States (excluding Croatia)	1997–2008	All-cause SMR for under 64 years of age	Member States	Sigma convergence	Interquartile and interdecile range, coefficient of variation, standard deviation of logs, Gini coefficient, Theil coefficient and Atkinson coefficient	Divergence in all 10 analyses

EU, European Union; LEB, life expectancy at birth; NUTS, nomenclature of territorial units for statistics; SMR, age- and sex-standardized mortality rate.

In relation to our secondary objectives, we also provide an overview of the sigma convergence dispersion measures used by the included studies (Supplementary table S4) and evaluate them using the following criteria: (i) the principle of transfers, (ii) the scale independence and (iii) the principle of population.^20^ We chose these three criteria because they reflect the potential of the measures to produce different results when used on the same dataset.

We examined the determinants of convergence that have been empirically evaluated in the included literature. This was possible for the determinants that were included as potential confounders in the models and the coefficients for each determinant explicitly reported. We did not include determinants that were not explicitly empirically evaluated, for example, if they were only discussed in the introduction or discussion sections of the included reports. We summarize the effect of the included determinants of convergence as being either positively or negatively associated with mortality convergence in table 2.

**Table 2:** Summary of the potential determinants of health convergence and of their effect on convergence

Determinant of health convergence	Maynou and Saez (2016), life expectancy	Maynou et al. (2015), life expectancy	Maynou and Saez (2016), mortality outcome
Country-level income inequality (Gini index)	Negative^a^	Negative	Negative
Gini index, 1-year lag	Positive	/	Positive^a^
Regional GDP per capita	None	Positive	Positive^a^
Regional GDP per capita (1-year lag)	None	Positive	Negative^a^
Regional GDP per capita (2-year lag)	Negative	Negative	Positive
Regional high-tech employment	Positive^a^	/	Positive
Regional university students	Negative	Negative	Negative
Regional secondary students	*/*	Negative	*/*
Regional youth male unemployment	Positive	Negative	Negative
Regional youth female unemployment	Positive	Positive	Positive
Country proportion GDP spent on R&D	Positive	/	Negative
Country external trade balance	Positive^a^	Positive^a^	Positive
Country public expenditure rate	Positive^a^	Negative	Negative^a^

*Note*: The effect is listed as positive and negative when differences in factors lead to higher and lower equilibrium states, respectively.

GDP, gross domestic product; R&D, research and development.

a The effect is reported as statistically significant.

### Risk of bias assessment

We assessed the risk of bias using a modified general framework introduced by Parmar et al.^27^ Their framework includes seven sources of potential bias: selection bias, ecological fallacy, confounding bias, reporting bias, time bias, measurement error in exposure and measurement error in outcome. We decided to evaluate the included studies on the selection bias, reporting bias and time bias domains. We considered the other four domains less appropriate for our review. We did not consider ecological fallacy because convergence is a macro-level phenomenon. We did not consider confounding bias because not including unit characteristics as potential confounders in the analysis is a legitimate choice and a test of absolute convergence as opposed to conditional convergence.^7^ We did not consider measurement error in exposure because our research question does not include an explicit exposure. Finally, we did not consider measurement error in health outcome because we included only health outcomes with very low likelihood of measurement error and there was no variation between the studies in this regard.

We conceptualized selection bias as present when studies excluded countries or regions due to a lack of data or perceived insufficient data quality, but absent when this was done for justifiable theoretical reasons (i.e. excluding overseas regions). We defined reporting bias as failing to report all aspects of the study (aims, methods or results were unclear). Finally, we defined time bias as considering an artificially short time period, i.e. the observation period starting long (>5 years) after 1990 and ending long before the publication date. We evaluated these domains using a binary score. A study was considered at high overall risk of bias if there was risk of bias in two or more of the domains, at medium risk of bias if there was risk of bias in one domain, and low risk of bias if there was no risk of bias in any of the domains.

## Results

### Study selection

**Figure 1:**
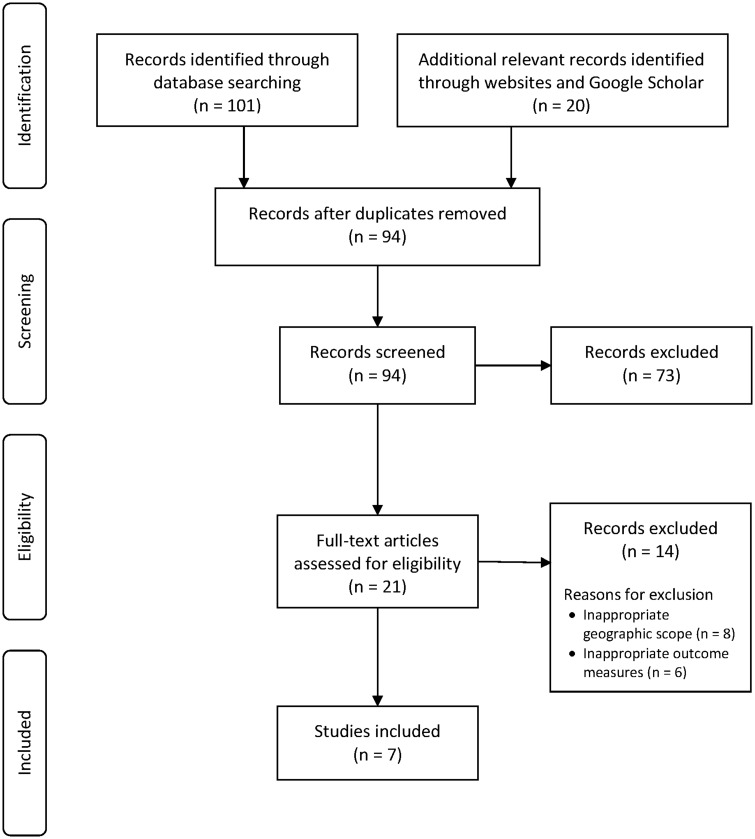
PRISMA flow diagram

Our search uncovered 121 records. Of these records, 101 were identified in electronic databases, four via searches of websites (all websites of EU institutions), and 16 via Google Scholar (figure 1). After performing deduplication and scanning the titles and summaries of the remaining 94 documents, we eliminated 73 records based on their titles and summaries. Finally, we scrutinized the full text of the remaining 21 records against the eligibility criteria, which eliminated a further 14 records. All 87 excluded records and the specific reasons for exclusion are shown in Supplementary table S3.

### Study characteristics

The seven included studies^28–34^ report 36 distinct analyses that feature various research designs and analytical approaches (table 1).

Five of the studies investigated convergence across the EU NUTS 2 regions (Nomenclature of Territorial Units for Statistics level 2),^28–31^^,^^34^ one examined convergence across the Member States^33^ and one included both the regions and the Member States as units of analysis.^30^ Richardson et al.^32^ studied convergence among the NUTS 2 regions grouped into deciles based on past mortality and income. While the time spans they covered differed substantially, the observation periods of all of the studies began before the 2004 enlargement, and the observation period of one study did not extend beyond the 2007 enlargement. Six studies included life expectancy at birth as the primary outcome measure, while two studies included age-standardized all-cause mortality rate, of which one includes only under 64 mortality. All of the included studies relied primarily on Eurostat data.

### Synthesis of results

**Figure 2:**
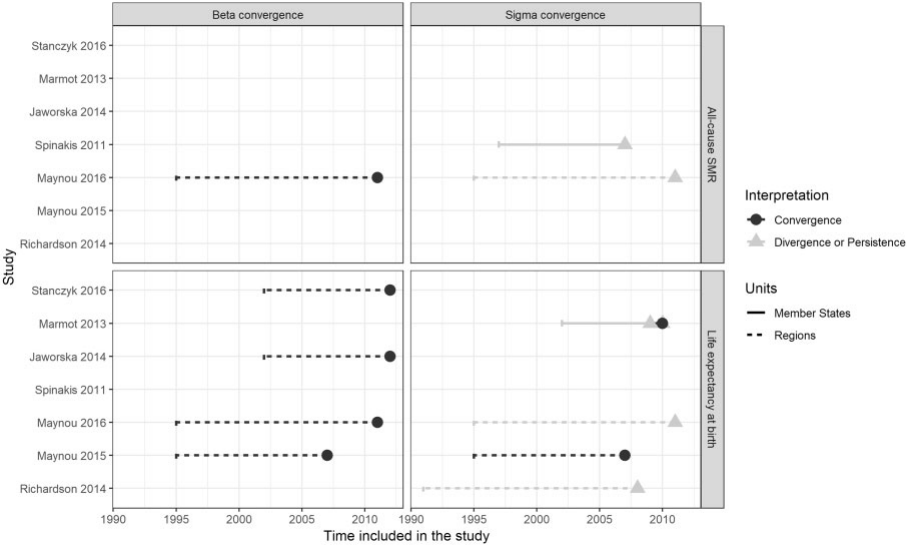
Summary of the results of the included studies against their design characteristics

There was almost perfect agreement when the results of the analyses were grouped by convergence concept (beta or sigma convergence). All of the analyses that relied on explicit beta convergence models^28^^,^^29^^,^^31^^,^^34^ found evidence of convergence, irrespective of the outcome measure. Of the 31 analyses that relied on the sigma convergence concept,^29–33^ 29 concluded that divergence occurred or that gaps between units persisted, irrespective of the unit of analysis, the outcome measure or the statistical dispersion measure used. The exceptions are Maynou et al.,^29^ who found evidence of sigma convergence when considering life expectancy at birth; and Marmot et al.,^30^ who found evidence of sigma convergence among the Member States when considering female life expectancy between 2000 and 2010. Figure 2 summarizes the interplay of the analytical approaches and the findings.

The five sigma convergence studies used a total of six different inequality measures: range (including interdecile range and interquartile range), standard deviation, coefficient of variation, the Gini index, the Theil index and the Atkinson index (Supplementary table S4). These measures differ in terms of the principle of transfers (i.e. whether they reflect all of the transfers from healthier units to less healthy units), and the scale independence (i.e. whether they are robust to proportionally equal changes to all units). These differences could lead them to produce different results when used on the same dataset, as two of the studies^30^^,^^33^ demonstrated. It should, however, be noted that the differences in the results did not produce qualitatively different conclusions.

None of the included studies explicitly aimed to investigate the potential determinants of mortality convergence. Two of the studies^29^^,^^31^ included a number of characteristics as potential confounders (table 2), but none of the country or regional factors were found to consistently point to the emergence of mortality convergence clubs when considering both life expectancy and all-cause mortality outcomes. Several factors were, paradoxically, found to be associated with both higher life expectancy and higher all-cause mortality or vice versa.

### Risk of bias

Supplementary table S5 summarizes the results of our risk of bias assessment. Most studies were considered at medium risk of bias, mostly due to the presence of the risk for time bias due to an unjustified selection of the observation period. Two studies^29^^,^^32^ were considered at high risk of bias.

## Discussion

Our systematic literature search on mortality convergence in the post-2004 EU uncovered 94 unique records. Seven studies that reported on 36 analyses were included. There was marked methodological heterogeneity across these studies, including differences in the convergence measures (beta and sigma convergence) used. All of the beta convergence analyses found evidence of convergence, whereas most of the sigma convergence analyses found evidence of divergence. These results are robust to the units of analysis and mortality and inequality measures. There is a lack of evidence on the determinants of health convergence in the EU in the studies included in this review.

The main result of our review is that the EU is experiencing beta convergence, but not sigma convergence. This finding suggests that although the regions and the Member States with high levels of initial mortality improved the fastest, this development did not lead to an overall reduction in mortality inequality between the geographic units. This result may seem paradoxical. Beta convergence is a necessary but not sufficient condition for sigma convergence.^7^ Two reasons why beta convergence and sigma divergence may occur together have been previously described. First, beta convergence might be a purely statistical artefact due to random fluctuation in the first or the last year of observation, since beta convergence is particularly susceptible to dynamics in the tails of the distribution.^19^ Second, a ‘change of role’ may have occurred that was accompanied by large improvements in units with very high initial mortality, whereas the areas with above average but less extreme initial mortality lost ground.^19^^,^^35^

Maynou and Saez,^31^ as well as others,^36^ observed that the 2008 economic crisis and the differential implementation of austerity may have exacerbated the differences in mortality across the EU. It was hypothesized that the effects have been the most negative in a group of western Member States that required financial assistance, particularly Greece, Spain and Portugal. While these countries have some of the highest life expectancies at birth in the EU, they experienced below average improvements between 2006 and 2011, whereas the eastern EU Member States experienced above average progress over the same period.^37^ These marked differences in performance at the tails of the distribution lend credence to the ‘change of role’ hypothesis discussed above.

On the other hand, some scholars have argued that beta convergence is simply a reflection of Galton’s fallacy of regression to the mean, and claim that sigma convergence is the only valid measure of convergence.^38^ The authors of two papers included in this review seem to agree with this argument, as the conclusions they provided were solely based on their sigma convergence analysis, even though they had also performed a beta convergence analysis.^29^^,^^31^ In our view, both instruments provide a valuable perspective on mortality convergence, and the final conclusion should be informed by a careful evaluation of the dynamics of the units in the tails of the distribution.

There was marked heterogeneity between the studies in terms of research design, and within the group of sigma convergence studies in terms of the inequality measures utilized. This finding suggests that an established best practice was lacking, or that there was a failure in its dissemination. Spinakis et al.^33^ concluded that ‘the Gini coefficient is the most appropriate solution for measuring health inequalities when the data refer to mortality, life expectancy and health expectancy rates’. It appears, however, that this recommendation has not been taken up by the other researchers in the field, with the exception of the Marmot et al.^30^ report.

The differences between the characteristics of the inequality measures could theoretically lead to different results when used to analyze the same data.^20^ This hypothesis was borne out in two of the studies.^30^^,^^33^ However, the qualitative conclusions were not changed by the choice of measure in the studies included in this review. Nevertheless, the popularity of simple inequality measures like range and standard deviation could be considered problematic in the light of their scale dependence, which means that a change in the range or the standard deviation could reflect improvements in mortality, rather than increases or decreases in the inequality of its distribution.^20^ Hence, despite being appealing because of the ease of their interpretation, these measures perform poorly as indicators of mortality convergence over the longer term, and should not be relied upon to evaluate the effect of policies in this area.

None of the included studies explicitly sought to test the determinants of health convergence. Two of the studies^29^^,^^31^ considered various regional- and country-level characteristics as covariates in their models of beta convergence. This approach could identify the characteristics that underlie the creation of convergence clubs, and might, therefore, act as a barrier to overall convergence. However, the results were contradictory and failed to clearly implicate any of the characteristics as barriers in the process of overall convergence. The remaining included studies did not attempt to explain the results of their convergence analyses. Since it is difficult to effectively argue for the importance of any factor in the mortality convergence or divergence trends identified without having a more complete understanding of its determinants, we presume that most of the authors decided against trying to provide such an explanation.

### Limitations

Our review might be biased due to our search strategy, selection procedure or publication bias. By using a three-part search strategy, we did our utmost to locate the relevant scientific documents. We tested for the possibility of selection bias by analysing a sample of studies that were excluded during the full-text screening stage and found that the results did not differ from those reported in the paper. We would argue that the risk of publication bias is low since any outcome in this context—i.e. the convergence, the divergence or the persistence of health disparities—is an interesting finding.

### Implications for research and policy

Efforts to harmonize approaches to measuring mortality convergence in the EU can lead to improved interpretability of the results, better surveillance of mortality convergence over time, and a higher likelihood of policy-makers acting on the evidence. We support the use of both beta and sigma convergence measures, that is, measures that compare the rate of growth between units with different starting points, and measures of inequality, respectively. We also recommend the use of inequality measures that meet the criteria of the strong principle of transfers and scale independence (e.g. Theil's entropy index) for tracking mortality convergence.

The lack of literature on the determinants of mortality convergence in the EU is an important research gap that leaves decision-makers without a sufficient evidence base to enact policies that effectively reduce geographic disparities in health. Constructing a framework of determinants of mortality convergence in the EU is, therefore, a key challenge for future research efforts in this field. Conducting in-depth research into countries and regions that have successfully bridged the east-west mortality gap, and comparing them with areas that continually fail to do so, could begin to fill this research gap. However, unlike in the existing research, the case selection in future studies should seek to identify samples that are representative of both the EU as a whole and of the range of mortality convergence trajectories that the EU Member States and regions experience. Developing novel methods of data-driven exploratory analysis, like clustering^39^ on area characteristics or mortality trajectories, could be helpful in identifying such samples.

The main implication of our results for policy-makers is that in their current form, the existing policies on regional cohesion and health inequalities do not seem to be effective in reducing geographic disparities in mortality. In particular, our findings show that although the most initially disadvantaged Member States and regions (the tail of the distribution) have likely improved the most, mortality improvements may have stagnated in the areas with moderate initial positions. This may be in part because the cohesion policy rules prioritize areas that currently have below average development indicators, without explicitly considering the trends in these indicators.^40^ A higher rate of mortality convergence could be achieved by providing more intensive support to communities that have historically stagnant rates of mortality improvement.

## Conclusion

Our systematic literature review has revealed that although the regions or the Member States with initially higher mortality rates improved faster than those with more favourable starting conditions, this trend did not lead to an overall reduction in dispersion across the units, and it may have even increased it. This seemingly paradoxical result might be explained by the negative impact of the 2008 economic crisis on mortality developments in the EU Member States with a recent history of low mortality rates (e.g. Greece, Spain and Portugal). Efforts to harmonize approaches to measuring mortality convergence and research into the determinants of mortality convergence are needed to better support evidence-informed policy aimed at reducing geographic disparities in mortality in the EU. While EU cohesion policy focuses on areas with below average outcomes in a cross-sectional perspective, it might also be necessary to focus on regions with historically stagnant rates of mortality improvement.

## Supplementary data

Supplementary data are available at *EURPUB* online.

*Conflicts of interest*: None declared.

**Key points**

- There is general agreement in the literature that the regions and the Member States with higher initial mortality rates improved the fastest, but that this trend did not lead to an overall convergence of mortality levels among geographic units in the EU.
- Given the heterogeneity in the research designs of mortality convergence studies and the susceptibility of popular inequality measures to secular trends in mortality, the standardization of approaches to measuring mortality convergence in the European Union (EU) that rely on both beta and sigma convergence concepts, and that utilize inequality measures that are robust to secular trends in the mean mortality rate, are needed.
- Given the current lack of evidence on the determinants of mortality convergence, we recommend a systematic study of well-chosen cases of mortality convergence or divergence to support the development of an evidence-based cohesion policy in the EU.
- Policy initiatives should support areas that experience moderate but stagnant mortality rates in addition to those with high mortality rates.

## Supplementary Material

ckaa038_supplementary_data
