# Supplementary material for: Mortality convergence in the enlarged European Union: a systematic literature review
Source: Eur J Public Health. 2020 Mar 24;30(6):1108–15. doi: 10.1093/eurpub/ckaa038 (PMC7733049; doi:10.1093/eurpub/ckaa038)
Supplement: ckaa038_supplementary_data [file ckaa038_supplementary_data.docx]

Mortality convergence in the enlarged European Union: a systematic literature review

# Supplementary material

Rok Hrzic, Tobias Vogt, Fanny Janssen, Helmut Brand

# Contents

Supplementary table 1. Eligibility criteria

Supplementary table 2. Keyword strategy

Supplementary table 3. List of excluded studies and the reason for exclusion (reverse chronological order)

Supplementary table 4. Dispersion measures included and their characteristics.

Supplementary table 5. Risk of bias assessment of included studies.

| **Supplementary table 1. Eligibility criteria** | | |
| --- | --- | --- |
| ***Domain*** | ***Inclusion criteria*** | ***Exclusion criteria*** |
| *Relevance* | Study explicitly aims to investigate health convergence or dynamics in geographic mortality inequalities in the EU. | Study presents general mortality or life expectancy trends without focusing on mortality convergence or dynamics in geographic mortality inequalities in the EU. |
| *Territory* | Study includes (or aims to include) the 25, 27, or 28 EU Member States after the 2004 enlargement. Studies may exclude some Member States or regions due to data availability or data quality concerns. | Study explicitly selects only a limited subset of EU Member States or regions or includes countries outside the current EU-28. |
| *Time of publication* | Document was accessible by May 2019, when the search was conducted. | None. |
| *Outcomes* | Document reports convergence in terms of standardised all-cause mortality rates (sex-specific or aggregate) or life expectancy at birth (sex-specific or aggregate). | Document reports convergence only in terms of outcomes that do not include all-cause mortality or life expectancy at birth. |
| *Convergence measure* | Report includes an explicit measure of convergence, and provides an interpretation of whether it found evidence of health convergence, health divergence, or stagnation of inequality based on that measure. | The document includes a conclusion on EU convergence without also reporting an explicit measure of convergence (e.g., the authors judge convergence or divergence in mortality trends from a graph only). |
| *Publication type* | Study report is in the form of a peer-reviewed scientific article, report, or working paper. | Study report is in the form of a monograph or doctoral dissertation. |

| **Supplementary table 2. Keyword strategy** | | |  |  |
| --- | --- | --- | --- | --- |
| **Geographic unit** | AND | **Phenomenon** | AND | **Measure** |
| European Union OR  European Economic Community OR  European Community |  | geographic* OR region* OR spatial  AND  ineq* OR disparit*  OR  converg* OR diverg* |  | mortality OR  life expectancy OR  lifespan OR  length of life |

| **Supplementary table 3. List of excluded studies and the reason for exclusion (reverse chronological order)** | |
| --- | --- |
| ***Study reference (in order of publication)*** | ***Reason for exclusion*** |
| 1. Viner RM, Ward JL, Wolfe I. Countdown for UK Child Survival 2017: mortality progress and targets. Archives of disease in childhood. 2018;103(5):474-9. | Not relevant to the research question. |
| 1. Ribeiro AI, Launay L, Guillaume E, Launoy G, Barros H. The Portuguese version of the European Deprivation Index: Development and association with all-cause mortality. PloS one. 2018;13(12):e0208320. | Not relevant to the research question. |
| 1. Nicholson A. Towards a more positive view of healthcare in Ireland. Irish journal of medical science. 2018. | Not relevant to the research question. |
| 1. Maher PJ, Igou ER, van Tilburg WAP. Brexit, Trump, and the Polarizing Effect of Disillusionment. Social Psychological and Personality Science. 2018;9(2):205-13. | Not relevant to the research question. |
| 1. Kuhmonen T. The Evolution of Problems Underlying the EU Agricultural Policy Regime. Sociologia Ruralis. 2018;58(4):846-66. | Not relevant to the research question. |
| 1. Kocourkova J, Sidlo L, Novak M, Sykora L. Growing gap in population dynamics, closing the gap in population size: the European Union and the United States compared. Geografie. 2018;123(1):37-62. | Not relevant to the research question. |
| 1. Eurofund. Upward convergence in the EU: Concepts, measurements and indicators. Luxembourg: Publications Office of the European Union; 2018. | Outcome measures do not include life expectancy at birth or adult all-cause mortality. |
| 1. Cleries R, Rooney RM, Vilardell M, Espinas JA, Dyba T, Borras JM. Assessing predicted age-specific breast cancer mortality rates in 27 European countries by 2020. Clinical & Translational Oncology. 2018;20(3):313-21. | Outcome measures do not include life expectancy at birth or adult all-cause mortality |
| 1. Chybalski F, Gumola M. The similarity of European pension systems in terms of OMC objectives: A cross-country study. Social Policy & Administration. 2018;52(7):1425-40. | Not relevant to the research question. |
| 1. Carracedo P, Debon A, Iftimi A, Montes F. Detecting spatio-temporal mortality clusters of European countries by sex and age. Int J Equity Health. 2018;17(1):38. | Study includes countries outside the scope of the EU-28. |
| 1. Bernal-Delgado E, Garcia-Armesto S, Oliva J, Sanchez Martinez FI, Repullo JR, Pena-Longobardo LM, et al. Spain: Health System Review. Health systems in transition. 2018;20(2):1-179. | Not relevant to the research question. |
| 1. Thomson KH, Renneberg AC, McNamara CL, Akhter N, Reibling N, Bambra C. Regional inequalities in self-reported conditions and non-communicable diseases in European countries: Findings from the European Social Survey (2014) special module on the social determinants of health. European Journal of Public Health. 2017;27:14-21. | Not relevant to the research question. |
| 1. Strandberg TE, von Bonsdorff M, Strandberg A, Pitkala K, Raikkonen K. Associations of vacation time with lifestyle, long-term mortality and health-related quality of life in old age: The Helsinki Businessmen Study. European Geriatric Medicine. 2017;8(3):260-4. | Not relevant to the research question. |
| 1. Richardson EA, Moon G, Pearce J, Shortt NK, Mitchell R. Multi-scalar influences on mortality change over time in 274 European cities. Soc Sci Med. 2017;179:45-51. | Study includes countries outside the scope of the EU-28. |
| 1. Ribeiro AI, Mayer A, Miranda A, de Pina MD. The Portuguese Version of the European Deprivation Index: An Instrument to Study Health Inequalities. Acta Medica Portuguesa. 2017;30(1):17-25. | Not relevant to the research question. |
| 1. Landsberg C, Hierro L. An overview of the EU-SA Strategic Partnership 10 years on: Diverging world views, persisting interests. South African Journal of International Affairs-Sajia. 2017;24(2):115-35. | Not relevant to the research question. |
| 1. Kashnitsky I, de Beer J, van Wissen L. Decomposition of regional convergence in population aging across Europe. Genus. 2017;73(1):2. | Not relevant to the research question. |
| 1. Elek P, Takacs E, Meresz G, Kalo Z. Implication of external price referencing and parallel trade on pharmaceutical expenditure: indirect evidence from lower-income European countries. Health Policy and Planning. 2017;32(3):349-58. | Not relevant to the research question. |
| 1. Debon A, Chaves L, Haberman S, Villa F. Characterization of between-group inequality of longevity in European Union countries. Insurance Mathematics & Economics. 2017;75:151-65. | Study does not report any measure of convergence. |
| 1. Carioli G, Negri E, Kawakita D, Garavello W, La Vecchia C, Malvezzi M. Global trends in nasopharyngeal cancer mortality since 1970 and predictions for 2020: Focus on low-risk areas. International Journal of Cancer. 2017;140(10):2256-64. | Study includes countries outside the scope of the EU-28. |
| 1. Bremberg SG. Mortality rates in OECD countries converged during the period 1990-2010. Scand J Public Health. 2017;45(4):436-43. | Study includes countries outside the scope of the EU-28. |
| 1. Ribeiro AI, Krainski ET, Carvalho MS, Pina Mde F. Where do people live longer and shorter lives? An ecological study of old-age survival across 4404 small areas from 18 European countries. J Epidemiol Community Health. 2016;70(6):561-8. | Outcome measures do not include life expectancy at birth or adult all-cause mortality |
| 1. Heggebo K. Health Effects of Unemployment in Denmark, Norway and Sweden 2007-2010: Differing Economic Conditions, Differing Results? International Journal of Health Services. 2016;46(3):406-29. | Not relevant to the research question. |
| 1. Hartley A, Marshall DC, Salciccioli JD, Sikkel MB, Maruthappu M, Shalhoub J. Trends in Mortality From Ischemic Heart Disease and Cerebrovascular Disease in Europe: 1980 to 2009. Circulation. 2016;133(20):1916-26. | Outcome measures do not include life expectancy at birth or adult all-cause mortality. |
| 1. Forsea AM. Cancer registries in Europe-going forward is the only option. Ecancermedicalscience. 2016;10. | Not relevant to the research question. |
| 1. Filippidis FT, Agaku IT, Vardavas CI. Geographic variation and socio-demographic determinants of the co-occurrence of risky health behaviours in 27 European Union member states. Journal of Public Health. 2016;38(2):E13-E20. | Not relevant to the research question. |
| 1. Ferreira P, Dionisio A. GDP growth and convergence determinants in the European Union: a crisp-set analysis. Review of Economic Perspectives. 2016;16(4):279-96. | Not relevant to the research question. |
| 1. Cuaresma JC, Loichinger E, Vincelette GA. Aging and income convergence in Europe: A survey of the literature and insights from a demographic projection exercise. Economic Systems. 2016;40(1):4-17. | Not relevant to the research question. |
| 1. Altobelli E, D'Aloisio F, Angeletti PM. Colorectal cancer screening in countries of European Council outside of the EU-28. World journal of gastroenterology. 2016;22(20):4946-57. | Not relevant to the research question. |
| 1. Yucesahin MM, Samir KC. Demographic and human capital heterogeneity in selected provinces of turkey: a scenario analysis using multi-dimensional population projection model. Economics & Sociology. 2015;8(3):215-44. | Not relevant to the research question. |
| 1. Tomeczkowski J, Lange A, Guntert A, Thilakarathne P, Diels J, Xiu L, et al. Converging or Crossing Curves: Untie the Gordian Knot or Cut it? Appropriate Statistics for Non-Proportional Hazards in Decitabine DACO-016 Study (AML). Advances in Therapy. 2015;32(9):854-62. | Not relevant to the research question. |
| 1. Seniori Costantini A, Gallo F, Pega F, Saracci R, Veerus P, West R. Population health and status of epidemiology in Western European, Balkan and Baltic countries. International journal of epidemiology. 2015;44(1):300-23. | Not relevant to the research question. |
| 1. Royuela V, Garcia GA. Economic and Social Convergence in Colombia. Regional Studies. 2015;49(2):219-39. | Not relevant to the research question. |
| 1. Rastorgueff PA, Bellan-Santini D, Bianchi CN, Bussotti S, Chevaldonne P, Guidetti P, et al. An ecosystem-based approach to evaluate the ecological quality of Mediterranean undersea caves. Ecological Indicators. 2015;54:137-52. | Not relevant to the research question. |
| 1. Newton JN, Briggs ADM, Murray CJL, Dicker D, Foreman KJ, Wang HD, et al. Changes in health in England, with analysis by English regions and areas of deprivation, 1990-2013: a systematic analysis for the Global Burden of Disease Study 2013. Lancet. 2015;386(10010):2257-74. | Not relevant to the research question. |
| 1. Marinho RT, Duarte H, Giria J, Nunes J, Ferreira A, Velosa J. The burden of alcoholism in fifteen years of cirrhosis hospital admissions in Portugal. Liver International. 2015;35(3):746-55. | Not relevant to the research question. |
| 1. Ebela I, Zile I, Mucina N, Razuka-Ebelal D, Rumba-Rozenfelde I. Territorial differences in infant mortality in Latvia in the first decade of the third millennium. Central European journal of public health. 2015;23(1):14-9. | Not relevant to the research question. |
| 1. Dell'Apa A, Bangley CW, Rulifson RA. Who let the dogfish out? A review of management and socio-economic aspects of spiny dogfish fisheries. Reviews in Fish Biology and Fisheries. 2015;25(2):273-95. | Not relevant to the research question. |
| 1. Costantini AS, Gallo F, Pega F, Saracci R, Veerus P, West R. Population health and status of epidemiology in Western European, Balkan and Baltic countries. International Journal of Epidemiology. 2015;44(1):300-23. | Not relevant to the research question. |
| 1. Chevreul K, Berg Brigham K, Durand-Zaleski I, Hernandez-Quevedo C. France: Health System Review. Health systems in transition. 2015;17(3):1-218, xvii. | Not relevant to the research question. |
| 1. Villaverde J, Maza A, Hierro M. Health care expenditure disparities in the European Union and underlying factors: a distribution dynamics approach. International Journal of Health Care Finance & Economics. 2014;14(3):251-68. | Not relevant to the research question. |
| 1. Taulbut M, Walsh D, McCartney G, Parcell S, Hartmann A, Poirier G, et al. Spatial inequalities in life expectancy within postindustrial regions of Europe: a cross-sectional observational study. BMJ Open. 2014;4(6):e004711. | Cross-sectional comparison, not dynamic in time. |
| 1. Song QK, Li J, Huang R, Fan JH, Zheng RS, Zhang BN, et al. Age of Diagnosis of Breast Cancer in China: Almost 10 Years Earlier than in the United States and the European Union. Asian Pacific Journal of Cancer Prevention. 2014;15(22):10021-5. | Not relevant to the research question. |
| 1. Mendes M. Comment on "Trends in age-specific coronary heart disease mortality in the European Union over three decades: 1980-2009". Revista Portuguesa De Cardiologia. 2014;33(1):65-6. | Not relevant to the research question. |
| 1. Innamorati M, Serafini G, Lester D, Amore M, Girardi P, Pompili M. Violent deaths among Russian and EU male older adults. International Journal of Social Psychiatry. 2014;60(1):89-94. | Not relevant to the research question. |
| 1. Castillo-Manzano JI, Castro-Nuno M, Fageda X. Can health public expenditure reduce the tragic consequences of road traffic accidents? The EU-27 experience. European Journal of Health Economics. 2014;15(6):645-52. | Not relevant to the research question. |
| 1. Robine J-M, Cambois E, Nusselder W, Jeune B, Oyen HV, Jagger C, et al. The joint action on healthy life years (JA: EHLEIS). Archives of public health = Archives belges de sante publique. 2013;71(1):2. | Not relevant to the research question. |
| 1. Nichols M, Townsend N, Scarborough P, Rayner M. Trends in age-specific coronary heart disease mortality in the European Union over three decades: 1980-2009. Eur Heart J. 2013;34(39):3017-27. | Outcome measures do not include life expectancy at birth or adult all-cause mortality. |
| 1. Mackenbach JP, Karanikolos M, McKee M. The unequal health of Europeans: successes and failures of policies. Lancet. 2013;381(9872):1125-34. | Study does not report any measure of convergence. |
| 1. Mackenbach JP, Hu Y, Looman CW. Democratization and life expectancy in Europe, 1960-2008. Soc Sci Med. 2013;93:166-75. | Not relevant to the research question. |
| 1. Mackenbach JP. Convergence and divergence of life expectancy in Europe: a centennial view. Eur J Epidemiol. 2013;28(3):229-40. | Study includes countries outside the scope of the EU-28. |
| 1. Mackenbach JP. Political conditions and life expectancy in Europe, 1900-2008. Soc Sci Med. 2013;82:134-46. | Study includes countries outside the scope of the EU-28. |
| 1. Hofmarcher MM, Quentin W. Austria: health system review. Health systems in transition. 2013;15(7):1-292. | Not relevant to the research question. |
| 1. Tilvis RS, Routasalo P, Karppinen H, Strandberg TE, Kautiainen H, Pitkala KH. Social isolation, social activity and loneliness as survival indicators in old age; a nationwide survey with a 7-year follow-up. European Geriatric Medicine. 2012;3(1):18-22. | Not relevant to the research question. |
| 1. Pornet C, Delpierre C, Dejardin O, Grosclaude P, Launay L, Guittet L, et al. Construction of an adaptable European transnational ecological deprivation index: the French version. Journal of Epidemiology and Community Health. 2012;66(11):982-9. | Not relevant to the research question. |
| 1. Mee JF. Reproductive issues arising from different management systems in the dairy industry. Reproduction in domestic animals = Zuchthygiene. 2012;47 Suppl 5:42-50. | Not relevant to the research question. |
| 1. Kossioni AE. Is Europe prepared to meet the oral health needs of older people? Gerodontology. 2012;29(2):e1230-40. | Not relevant to the research question. |
| 1. Kaitelidou D, Mladovsky P, Leone T, Kouli E, Siskou O. Understanding the oversupply of physicians in greece: the role of human resources planning, financing policy, and physician power. International Journal of Health Services. 2012;42(4):719-38. | Not relevant to the research question. |
| 1. Emelyanova A, Rautio A. Aging population of the Barents Euro-Arctic Region. European Geriatric Medicine. 2012;3(3):167-73. | Not relevant to the research question. |
| 1. Anell A, Glenngard AH, Merkur S. Sweden health system review. Health systems in transition. 2012;14(5):1-159. | Not relevant to the research question. |
| 1. Rehm J, Zatonksi W, Taylor B, Anderson P. Epidemiology and alcohol policy in Europe. Addiction. 2011;106:11-9. | Not relevant to the research question. |
| 1. Mohangoo AD, Buitendijk SE, Szamotulska K, Chalmers J, Irgens LM, Bolumar F, et al. Gestational age patterns of fetal and neonatal mortality in Europe: results from the Euro-Peristat project. PloS one. 2011;6(11):e24727. | Not relevant to the research question. |
| 1. Bonneux LG, Huisman CC, de Beer JA. Mortality in 272 European regions, 2002-2004. An update. European Journal of Epidemiology. 2010;25(2):77-85. | Cross-sectional comparison, not dynamic in time. |
| 1. Arnold M, Razum O, Coebergh J-W. Cancer risk diversity in non-western migrants to Europe: An overview of the literature. European journal of cancer (Oxford, England : 1990). 2010;46(14):2647-59. | Not relevant to the research question. |
| 1. Priemus H, Schutte-Postma E. Notes on the Particulate Matter Standards in the European Union and the Netherlands. International Journal of Environmental Research and Public Health. 2009;6(3):1155-73. | Not relevant to the research question. |
| 1. Hedrich D, Pirona A, Wiessing L. From margin to mainstream: The evolution of harm reduction responses to problem drug use in Europe. Drugs-Education Prevention and Policy. 2008;15(6):503-17. | Not relevant to the research question. |
| 1. Zatonski WA, Manczuk M, Powles J, Negri E. Convergence of male and female lung cancer mortality at younger ages in the European Union and Russia. European Journal of Public Health. 2007;17(5):450-4. | Outcome measures do not include life expectancy at birth or adult all-cause mortality. |
| 1. Happich M, von Lengerke T. Convergence of life expectancy in the European Union: a Markov approach. Applied Economics Letters. 2007;14(3):175-8. | Study does not include the full scope of the enlarged EU. |
| 1. Ostergren MM, Rasmussen VB. Child and adolescent health in a European perspective--a new WHO-strategy. Ugeskrift for laeger. 2006;168(36):3024-6. | Not relevant to the research question. |
| 1. Judge K, Platt S, Costongs C, Jurczak K. Health Inequalities: a Challenge for Europe. UK Presidency of the EU 2005; 2006. | Not relevant to the research question. |
| 1. De Beer J. Future trends in life expectancies in the European Union. Brusssels: European Commission Directorate-General "Employment, Social Affairs and Equal Opportunities"; 2006. | Study does not include the full scope of the enlarged EU. |
| 1. Berentsen WH. Changing regional inequalities in United Germany. Eurasian Geography and Economics. 2006;47(4):462-77. | Not relevant to the research question. |
| 1. Stewart K. Dimensions of well-being in EU regions: Do GDP and unemployment tell us all we need to know? Social Indicators Research. 2005;73(2):221-46. | Not relevant to the research question. |
| 1. Noin D. Spatial inequalities of mortality in the European Union. Europe’s Population: Routledge; 2005. p. 60-70. | Study does not include the full scope of the enlarged EU. |
| 1. Levi F, Bosetti C, Lucchini F, Negri E, La Vecchia C. Monitoring the decrease in breast cancer mortality in Europe. Eur J Cancer Prev. 2005;14(6):497-502. | Outcome measures do not include life expectancy at birth or adult all-cause mortality. |
| 1. van Doorslaer E, Koolman X. Explaining the differences in income-related health inequalities across European countries. Health Economics. 2004;13(7):609-28. | Not relevant to the research question. |
| 1. Treffers PE. Forty years of discussion about perinatal mortality in the Netherlands. Nederlands tijdschrift voor geneeskunde. 2004;148(38):1853-5. | Outcome measures do not include life expectancy at birth or adult all-cause mortality. |
| 1. Prioux F. The recent demographic evolution of France. Population. 2003;58(4-5):589-622. | Outcome measures do not include life expectancy at birth or adult all-cause mortality. |
| 1. European Commission. The health status of the European Union - Narrowing the health gap. Luxembourg: Office for Official Publications of the European Communities; 2003. | The document does not explicitly report the data regarding the gaps between the Member States in terms of life expectancy or adult all-cause mortality. |
| 1. Santana P. Poverty, social exclusion and health in Portugal. Social Science & Medicine. 2002;55(1):33-45. | Not relevant to the research question. |
| 1. McMurray DN. A coordinated strategy for evaluating new vaccines for human and animal tuberculosis. Tuberculosis. 2001;81(1-2):141-6. | Not relevant to the research question. |
| 1. Shaw M, Orford S, Brimblecombe N, Dorling D. Widening inequality in mortality between 160 regions of 15 European countries in the early 1990s. Social Science & Medicine. 2000;50(7-8):1047-58. | Study does not include the full scope of the enlarged EU. |
| 1. Mackenbach JP, Cavelaars A, Kunst AE, Groenhof F, Inequ EUWGS. Socioeconomic inequalities in cardiovascular disease mortality - An international study. European Heart Journal. 2000;21(14):1141-51. | Not relevant to the research question. |
| 1. Micklewright J, Stewart K. Is the well-being of children converging in the European Union? Economic Journal. 1999;109(459):F692-F714. | Not relevant to the research question |
| 1. Martinez-Gonzalez MA, Martinez JA, Hu FB, Gibney MJ, Kearney J. Physical inactivity, sedentary lifestyle and obesity in the European Union. International Journal of Obesity. 1999;23(11):1192-201. | Not relevant to the research question. |
| 1. Van Hoorn WD, De Beer J. Analysis and projection of national and regional mortality for countries of the European Economic Area. Maandstatistiek van de bevolking (Hague, Netherlands : 1982). 1998;98(6):8-16. | Not relevant to the research question. |
| 1. Neukirch F, Maguin P, Perdrizet S, Pariente R. Validity of mortality data in respiratory diseases in France and 7 other countries of the E.E.C. Revue des maladies respiratoires. 1984;1(6):361-7. | Not relevant to the research question. |

| **Supplementary table 4. Dispersion measures included and their characteristics.** | | | | |
| --- | --- | --- | --- | --- |
| ***Measure of dispersion*** | ***Definition*** | ***Principle of transfers*** | ***Scale independence*** | ***Principle of population*** |
| Range, including inter-decile range and inter-quartile range | Absolute difference between the unit with the highest and the unit with the lowest value. | Fails | No | Yes |
| Standard deviation | Average distance from the mean. | Strong | No | Yes |
| Coefficient of Variation | Ratio of standard deviation to the mean. | Weak | Yes | Yes |
| Gini index | Average distance between all units in the population as a proportion of the total. | Weak | Yes | Yes |
| Theil index | Entropy-based inequality measures. | Strong | Yes | Yes |
| Atkinson index |  | Weak | Yes | Yes |

| **Supplementary Table 5. Risk of bias assessment of included studies.** | | | | |
| --- | --- | --- | --- | --- |
| ***Study*** | ***Selection bias*** | ***Reporting bias*** | ***Time bias*** | ***Overall risk of bias*** |
| **Stańczyk, 2016** | Absent | Absent | Present | Medium |
| **Maynou et al., 2016** | Absent | Present^1^ | Absent | Medium |
| **Maynou et al., 2015** | Absent | Present^1^ | Present | High |
| **Richardson et al., 2014** | Present^2^ | Present^3^ | Present | High |
| **Jaworska, 2014** | Absent | Absent | Present | Medium |
| **Marmot et al., 2013** | Absent | Absent | Present | Medium |
| **Spinakis et al., 2011** | Absent | Absent | Present | Medium |
| Note: A study was considered at high risk of bias if two or more of the domains were positive, at medium risk of bias if one domain was positive, and low risk of bias if none were positive.  ^1^ The results of the sigma convergence analysis were reported only as plots, which made it impossible to extract precise numerical results.  ^2^ Most Eastern European Member States were excluded without justification.  ^3^ Most results were reported only as plots, which made it impossible to extract precise numerical results. | | | | |
